# Cofilin(s) and Mitochondria: Function Beyond Actin Dynamics

**DOI:** 10.3390/ijms26094094

**Published:** 2025-04-25

**Authors:** Tatiana Kovaleva, Murat Gainullin, Irina Mukhina, Vladimir Pershin, Liudmila Matskova

**Affiliations:** 1 Institute of Fundamental Medicine, Privolzhsky Research Medical University, 10/1 Minin Sq., 603005 Nizhny Novgorod, Russia; mukhinaiv@mail.ru (I.M.); bp1995@yandex.ru (V.P.); 2 Oslo University Hospital, N-0424 Oslo, Norway; murat.r.gainullin@gmail.com; 3 Microbiology and Tumor Biology Center (MTC), Karolinska Institutet, Solnavägen 9, Q8C, 17165 Stockholm, Sweden; 4 Institute of Molecular Biology and Biophysics, Federal Research Center of Fundamental and Translational Medicine (IMBB FRC FTM), 2/12, Timakova Street, 630117 Novosibirsk, Russia

**Keywords:** cofilin, lipid metabolism, K63 ubiquitination, mitochondria, neurodegeneration, tumorigenesis

## Abstract

ADF/cofilins form a family of small, widely expressed actin-binding proteins, regulating actin dynamics in various cellular and physiological processes in all eukaryotes, from yeasts to animals. Changes in the expression of the ADF/cofilin family proteins have been demonstrated under various pathological conditions. The well-established role of cofilin in migration, invasion, epithelial-mesenchymal transition, apoptosis, resistance to radiotherapy and chemotherapy, immune escape, and transcriptional dysregulation in malignant tumors is primarily attributed to its actin-modifying activity. Moreover, drugs targeting this function of cofilin have been developed for cancer treatment. However, its multilevel regulation, highly diverse effects across various pathological conditions, and conflicting data on the functional consequences of altered cofilin expression have prompted us to explore additional roles of cofilin—beyond actin modulation—particularly its involvement in lipid metabolism and mitochondrial homeostasis. Here, we review recent data on the expression of ADF/cofilin family proteins in various pathologies, account for the mutations and post-translational modifications of these proteins and their functional consequences, dwell on the role of K63-type ubiquitination of cofilin for its involvement in lipid metabolism and mitochondrial homeostasis, more specifically, a process of mitochondrial division or mitofission, point out conflicting data in cofilin research, and describe prospects for future studies of cofilin functions.

## 1. Introduction

Mitochondria are dynamic organelles, and permanent fission and fusion are essential to maintain their function in energy metabolism, calcium homeostasis, regulation of reactive oxygen species (ROS) and apoptosis [1]. The process of mitochondrial fission, its mechanics, and the factors involved in this process are increasingly coming into focus as a causative factor in oncogenic cell transformations and neurodegeneration. It is now becoming clear that mitochondrial fission can drive both pathological processes, i.e., promoting the survival of transformed, neoplastic cells, and ensure homeostasis of normal cells, providing energy and protection from oxidative stress. Whether it depends on specific cellular factors, their interactions, and/or the rate of this process requires further research. It is very important to characterize as many cellular factors involved in mitochondrial dynamics as possible, under different cellular conditions and stimuli. Cofilin is one such cellular factor [2,3,4,5].

**Figure 1:**
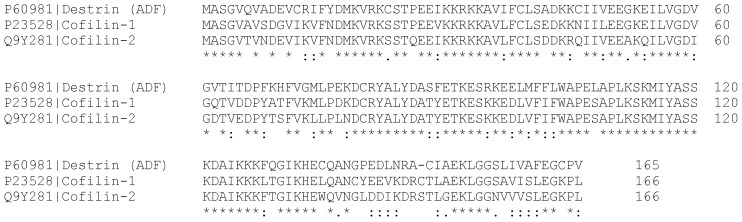
Structural alignment of the amino acid sequences of cofilin isoforms [18,19,20]. All three proteins from the cofilin family consist entirely of a single globular ADF homology domain (ADF-H), but several of their amino acids are different (labeled by a colon or a dot), sharing >70% sequence identity within an organism (labeled with asterisks). The nuclear localization signal is located at the N-terminus (at positions 30–40), followed by cysteines forming intramolecular bonds at positions C39–80, C139–147. These disulfide bonds serve as a mitochondrial targeting signal for cofilin. In the middle of the cofilin sequence and further on, closer to the C-terminus, there are nine residues scattered over a rather extended sequence (58 aa) responsible for binding filamentous actin (F-actin); four of them at this site, closer to the C-terminus, also bind globular actin (G-actin) [21].

The ubiquitous cellular protein cofilin attracted the interest of researchers almost 50 years ago [6]. Since then, several cofilin homologues were identified. ADF/cofilins are a family of small, widely expressed actin-binding proteins, regulating actin dynamics in various cellular and physiological processes in all eukaryotes, from yeasts to animals [7,8,9,10]. More precisely, cofilin depolymerizes filamentous actin (F-actin), producing monomeric, globular actin (G-actin). Mammals express all three members of the ADF/cofilin family: cofilin-1, cofilin-2, and ADF (actin depolymerizing factor, also called destrin) [7]. Non-muscle cells and tissues mostly express cofilin-1 and ADF, but their expression level may vary. Certain cell types express all three ADF/cofilins [11,12]. Cofilin-2 is found primarily in muscle [13], but also in brain and liver [14], oligodendrocytes, and keratinocytes [11,12]. While the three ADF/cofilin proteins share some overlapping functions, each performs unique functions in vivo. GWAS data analysis performed separately for cofilin-1, cofilin-2, and ADF, shows that all three proteins are associated with completely different phenotypes [15]. Mice deficient in cofilin-1 display early embryonic lethality and defects in actin-dependent morphogenic processes [16]. Such mutants are also lethal in yeast. ADF inactivation leads to corneal defects [16,17]. Although ADF and cofilin-1 share 70% of sequence identity and some overlapping functions, cofilin-1 is the major non-muscle isoform of ADF/cofilin in various cell types [11] (Figure 1).

In the molecular structure of cofilin, two actin-binding sites are present. One site binding both monomeric (G) and filamentous (F) actin, the second site interacts only with F-actin. Binding of cofilin to the actin filament causes a change in the orientation of actin subunits, which results in actin filament severing [8].

Cofilin-1 expression is regulated by microRNAs (miRNAs), as recently reviewed [22]. MiR-342 targets cofilin in human breast cancer cells, miR-429 targets cofilin in colon cancer cells, and miR-182-5p binds to cofilin mRNA in human bladder cancer cells. miR-134 was reported to suppress translation of cofilin [23]. Other miRNAs, such as miR-138 and miR-384, modulate the activity and expression of cofilin in ovarian cancer [24] and esophageal squamous cell carcinoma by targeting LIMK1 kinase [25]. These miRNAs all act as inhibitors of cofilin activity either by directly targeting cofilin or its upstream effector, LIMK1 kinase.

Changes in the expression of the ADF/cofilin family proteins have been demonstrated under various pathological conditions. Mutations in the cofilin-2 gene can cause a variety of pathologies in different organisms. In humans, it was shown to cause myopathies [26]. In mice, its deficiency causes disruptive accumulation of F-actin in skeletal muscles [9] and abnormalities of the sarcomeric architecture. The cofilin-1 gene is overexpressed in metabolic syndrome in humans [27] and may be involved in neurodegeneration, as was demonstrated in Aplysia [28]. Cofilin activity was found to be changed in Alzheimer’s, Parkinson’s, and Huntington’s diseases, spinal muscular atrophy, amyotrophic lateral sclerosis, prion diseases, and deletion-duplication syndromes [29]. The mRNA levels and expression of cofilin-1 was higher in most tumor tissues, as compared to normal in various types of cancer, such as non-small cell lung cancer, prostate cancer, vulvar squamous cell carcinoma, hepatoblastoma, breast cancer, ovarian cancer, and bladder cancer [30,31,32]. At the same time, an increased methylation level of the cofilin-1 promoter regions was present in colon and rectal adenocarcinoma tissues, according to the TCGA database [33,34]. The overexpression of cofilin was shown to be correlated with proliferation, invasion, metastasis, and poor survival. However, in esophageal carcinoma, tumor size, infiltration depth, and patient age were not found to be associated with the expression level of cofilin. Instead, cofilin expression correlated with various degrees of tumor differentiation, lymph node metastasis, and clinical stages [35]. In prostate cancer, there was no association between the expression of cofilin-1 and other clinicopathological variables, such as age and pathological stage. Downregulation of the cofilin-1 gene expression increases the percentage of apoptotic cells in the T24 and RT4 bladder cancer cell lines [32]. The established role for cofilin in migration, invasion, epithelial-mesenchymal transition (EMT), apoptosis, radiotherapy and chemotherapy resistance, immune escape, and transcriptional dysregulation of malignant tumors [22] is explained mainly by the cofilin-controlled mechanic activity of cells, like proliferation [36], cell migration [16,36,37,38,39,40], cell adhesion [41,42], and colony formation [31]. With this in mind, the cofilin gene might become a novel target in the strategy of diagnosis and even treatment of cancer, which warrants a careful study of all aspects of cofilin activity.

Details of derailed cofilin signaling, which leads to actin filament severing, depolymerization, nucleation, and bundling, are currently under active investigation. The initial understanding that cofilin depolymerizes actin fibers has now been refined. Cofilin exerts its highest actin severing activity when the cofilin:actin ratio is around 1:800 [43]. When the cofilin:actin ratio is higher than that, i.e., when more cofilin molecules are expressed, cofilin stabilizes F-actin, in which all subunits have undergone cofilin-induced rotation [43]. Cofilin can also induce nucleation of actin. Inactive, phosphorylated cofilin (p-cofilin) does not significantly bind to F-actin, and its actin severing or depolymerization activity is low [44].

Interestingly, although elevated cofilin expression is generally associated with increased cell motility [16,30,31,32,45], glioblastoma cells overproducing cofilin have decreased motility as compared to cells producing a moderate amount of cofilin [46]. The ADF/cofilin complex is accumulated in confluent cells, and this causes G1 phase arrest in the cell cycle progression in a variety of cell lines [47]. It is possible that this effect is mediated by other cofilin functions independent of its involvement in cytoskeleton reorganization [22] and may be modulated by its multiple post-translational modifications and multiple interacting cellular factors.

## 2. Post-Translational Modifications of Cofilin

Post-translational modifications (PTMs) play important roles in regulating cofilin-1 function by allowing local control for enhanced versatility. Thus, the same ubiquitous cytoplasmic protein cofilin is involved in a multitude of cellular processes, sensing local pH, oxidative stress, and others [48,49], being spatiotemporally orchestrated by numerous extra- and intra-cellular factors [22]. The multitude of PTMs of cofilin, such as phosphorylation [50], acetylation [51], ubiquitination [37], S-nitrosylation [52], ISG15-ylation, etc., and their combinations [44], in addition to the various expression levels of cofilin, is probably what allows this protein to transmit diverse signals to the cellular environment in very precise ways. Phosphorylation is a major type of PTM, and it is also the best studied. It regulates a variety of cellular signaling pathways in control of cell growth, division, differentiation, motility, and cell death [53].

**Figure 2:**
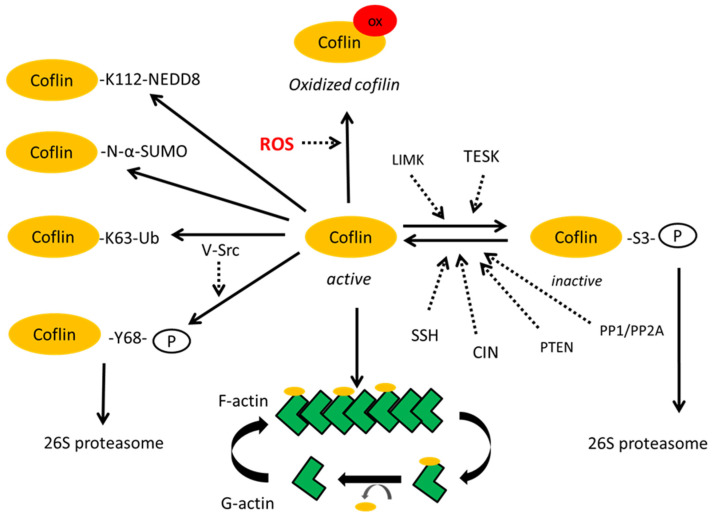
Regulation of cofilin activity. Cofilin is phosphorylated at the S3 position by the LIM and TES kinases [54]. Chronophin (CIN), Slingshot phosphatase (SSH) and tensin homolog (PTEN), and serine/threonine phosphatases type 1 (PP1) and type 2A (PP2A) dephosphorylate cofilin at the S3 position and activate cofilin, leading to depolymerization of filamentous actin (F-actin) [56]. Reactive oxygen species (ROS) lead to oxidation of cofilin [48,49]. Phosphorylation of cofilin at Y68 triggers degradation of cofilin via the ubiquitin-proteasome pathway [59]. Cofilin can be modified by ubiquitin and ubiquitin-like proteins (SUMO or NEDD8) [65].

The best studied PTM of cofilin is its phosphorylation. Cofilin is phosphorylated at the Serine 3 (S3) position by the LIM and TES kinases [54]. They can indirectly control filamentous (F-actin) stability through changing the level of cofilin phosphorylation, thereby decreasing the stability of active cofilin, as their overexpression in cells leads to F-actin accumulation [41,55]. Chronophin (CIN) and Slingshot (SSH) are specific cofilin phosphatases that dephosphorylate cofilin at the S3 position, thus protecting it from degradation [56]. The more generic serine/threonine phosphatases type 1 (PP1) and type 2A (PP2A) have also been reported to dephosphorylate cofilin [45]. Moreover, it was established that phosphatase and tensin homolog (PTEN) can directly dephosphorylate and activate cofilin-1, leading to depolymerization of F-actin [57] (Figure 2).

Additionally, cofilin is phosphorylated at Threonine 63 (T63), Tyrosine 82 (Y82), and S108 [58]. Phosphorylation of Y68 and Y140 has also been demonstrated [59]. Phosphorylation at Y68 triggers degradation of cofilin via the ubiquitin–proteasome pathway and, consequently, counteracts the cellular functions of cofilin in reducing cellular F-actin contents and cell spreading. Another example of cross-regulated post-translational modification of cofilin is neddylation-stimulated phosphorylation [60]. Cross-regulation serves as a general mechanism of post-translational modification [61,62,63].

Like other cellular molecules which participate in inactivation of ROS (glutathione, lipoic acid, thioredoxin), cofilin contains several thiol (SH) groups which, under conditions of oxidative stress, mediate oxidation of cysteine (C) residues leading to the appearance of cofilin dimers due to formation of disulfide bridges, which can cross-link actin filaments. Stable actin-cofilin rods save cellular ATP, which is not used during the active polymerization process. This facilitates faster cell recovery from stress. The intermolecular disulfide bonds mediate formation of dimers, trimers, and oligomers of cofilin [64].

Ubiquitin modifications of cofilin are not fully understood, especially in terms of specifying which specific lysine (K) in cofilin can covalently bind ubiquitin molecules in specific cells under specific conditions. Recently, a lysine-less mutant cofilin-1^25KR^ was created where all lysine was mutated [65].

It was shown that K112 of cofilin binds NEDD8, a ubiquitin-like molecule [60]. The lysines K45,53,144,164 [47] have been suggested as additional sites of cofilin ubiquitination [66]. Obviously, the proximity of the ubiquitination and phosphorylation sites suggests that modification may take place competitively. However, to resolve this question, a 3D structure will be required.

Thus, modifications of cofilin, especially the ubiquitination and the sequential coupled modifications (for example, phosphorylation and ubiquitination and vice versa), still await exploration at the molecular level to gain further insights in the dynamic regulation of cofilin activity.

Of the many PTMs of cofilin, the phosphorylation at S3 position is of key importance since it generates inactive phospho-cofilin, which is recognized as a degradation signal by the proteasome system.

The significance of PTMs of cofilin unrelated to its degradation still awaits further exploration. The K63 ubiquitin-branched modification of cofilin is one such PTM, probably mediated by the AIP4 ubiquitin ligase [38]. The K63 ubiquitinated proteins usually mediate the formation of inducible protein complexes that convey a variety of signals depending on the protein composition in the complexes [67,68,69].

The use of mass spectrometry-based proteomics has greatly accelerated the discovery of new PTMs and their sites of action on various proteins [70]. The most recent cofilin-1 modification reported describes N-terminal α-amino SUMOylation of cofilin-1, which is critical for its regulation of actin depolymerization [65].

However, cofilin-mediated actin dynamics can drive motility without post-translational regulation [8,65].

## 3. The Mitochondrial Localization of Cofilin

The localization of cofilin in mitochondria was reported several years ago, when its increased expression was noticed in connection with the Warburg effect in tumor cells [71]. In the following years, many papers have documented the translocation of cofilin to mitochondria upon treatments that initiate apoptotic or necrotic cell death, as recently reviewed [8].

Moreover, regions in cofilin were identified, which are critical for mitochondrial localization (in mammalian cells, specific amino acids at position 15–30 at the N-terminus and at position 106–166 at the C-terminus), which suggests that cofilin-1 indeed can bind to mitochondria directly [72]. It is therefore perplexing that cofilin is still not included in the Inventory of Mammalian Mitochondrial Proteins [73].

Since then, the functional consequences of cofilin involvement in mitochondrial dysfunction has become a focus of active studies as cofilin affects many aspects of mitochondrial homeostasis. Cofilin found in the mitochondrial fraction of cells was characterized as unphosphorylated [72,74], oxidized [48,72], or modified with the K63 branched ubiquitin chains [75]. In general, cofilin expression in pathological conditions has been associated with mitochondrial dysfunction, mediating cell death or cell division.

Most of the published data up to now on the association of cofilin with mitochondria attribute the cofilin-mediated effect on mitochondria and, in general, on cells, mainly, to actin reorganization. Cofilin controls mitochondrial traffic along the microtubules and actin [8]. Cofilin regulates mitochondrial morphology and function via redistribution of phospho-cofilin, cofilin, and its ubiquitinated proteoforms between the cytoplasm and mitochondria. This has been shown to correlate with changes in tissue respiration activity and mitophagy in mouse brain nerve cells [75].

Mitochondria are dynamic organelles, and permanent fission and fusion are essential to maintain their function in energy metabolism, calcium homeostasis, regulation of ROS, and apoptosis. Under oxidative stress, active and oxidized cofilin can be translocated into the mitochondria [76].

**Figure 3:**
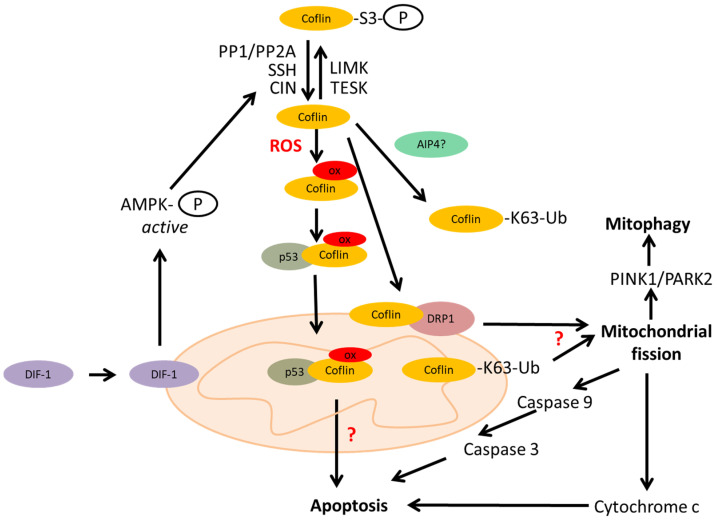
Role of cofilin in mitochondria metabolism. LIM and TES kinases phosphorylate and inactivate cofilin [54]. Cofilin is activated by chronophin (CIN), Slingshot (SSH), and serine/threonine phosphatases type 1 (PP1) and type 2A (PP2A) [56]. Differentiation-inducing factor 1 (DIF-1) activates chronophin via AMP-activated protein kinase (AMPK) [79]. The K63 ubiquitin-branched modification of cofilin is likely mediated by the AIP4 ubiquitin ligase [38]. Under oxidative stress, active and oxidized cofilin can be translocated into the mitochondria [72,74]. Cofilin recruits the dynamin-related protein 1 (DRP1) [77,78] to mitochondria. Cofilin potentiates mitochondrial fission as well as PINK1/PARK2-dependent mitophagy [4]. Mitochondrial fission may lead to the release of cytochrome c and activation of caspase resulting in apoptosis. Apoptosis can also be initiated by the cofilin/p53 pathway [76].

By regulating the actin cytoskeleton, cofilin induces mitochondrial fission, the first step in mitophagy. The molecular mechanism of cofilin-induced mitochondrial fission is being elucidated. First of all, cofilin itself is recruited to mitochondria in the mitochondrial fission process [2,3,4,5]. Cofilin recruits the dynamin-related protein 1 (Drp1), a key factor in the mitochondrial fission machinery [77,78], to mitochondria. Cofilin is activated in this process by differentiation-inducing factor 1 (DIF-1) as it activates pyridoxal phosphatase (or CIN) via AMP-activated protein kinase (AMPK). Cofilin can be activated by two other phosphatases: by PP1/PP2A via Src-Akt-mTOR and PTEN-PI3K pathways, and by SSH. Cofilin knockdown inhibits mitochondrial fission and decreases the protein levels of mitofusin 2 (MFN2), a crucial factor required for mitophagy [79]. Cofilin potentiates mitochondrial fission as well as PINK1/PARK2-dependent mitophagy [4,80]. Mitochondrial fission may activate the release of cytochrome c and caspase-9, resulting in apoptosis. Moreover, cofilin-1 was shown to participate directly in the opening of the mitochondrial permeability transition pore and releasing cytochrome c, leading to apoptosis progression, independent of its role in mitochondrial dynamics [49]. Apoptosis can also be initiated by the cofilin/p53 pathway [76] (Figure 3).

Thus, it has now become clear that cofilin, in addition to its actin depolymerizing activity, also affects several metabolic functions. Interestingly, in response to environmental challenges, cofilin uses its actin depolymerizing activity and mitochondria-coupled activity independent from each other [81]. Cofilin localizes on the mitochondria and enters the mitochondria. The functional consequences of these processes are under active investigation. One of the most speculative issues discussed currently is how actin enters into mitochondria and the role it plays there [8]. Cofilin may serve to deliver G-actin into mitochondria.

## 4. Cofilin Mediated Mitochondrial Dysfunction During Neurodegeneration

Effects of deranged cofilin activity on mitochondria were reported in neurodegenerative diseases like Parkinson’s and Alzheimer’s disease (AD) [28], and were characterized by neuronal degeneration and death, as well as by distorted synapse formation, oxidative stress, etc. [3,11,44,76].

This deranged cofilin activity mainly resulted in the generation of abnormal cytoplasmic structures. In neurons, these structures caused abnormal distribution of cellular organelles, such as mitochondria or early endosomes, loss of pre- and postsynaptic compartments, and therefore, reduced synaptic transmission and impaired neuronal plasticity [82]. A complex relationship between mitochondrial function (ATP synthesis, ROS level, autophagy) and cofilin ubiquitination was shown in the nerve cells [83].

In Parkinson’s disease (PD) cofilin-1 binds to α-synuclein and promotes its aggregation. These aggregates are observed at the onset and progression of PD. Cofilin facilitates the prion-like transmission of α-synuclein-fibrils into neurons [84]. Apart from general distortion of cytoskeletal organization by these aggregates, these structures cause mitochondrial dysfunction [85].

In Alzheimer’s disease, which is characterized by proteinopathies like rod shaped actin bundles (rods), amyloid-β (Aβ) peptide, and hyperphosphorylated tau, cofilin translocates to mitochondria inducing neurotoxicity. Activated cofilin (not phosphorylated at the S3 position) acts as a bridge between actin and microtubule dynamics by displacing tau from microtubules, thereby destabilizing tau-induced microtubule assembly, mis-sorting tau, and promoting tauopathy [86]. K63-dependent ubiquitination of cofilin was suggested to influence the level of cofilin, autophagy activation, actin dynamics, and bundle organization in the nerve cells [87].

Under AD pathological conditions, cellular ROS causes oxidation of cysteine residues of cofilin at positions 39, 80, 139, and 147 and of the methionine residue at position 115. At the same time, the four cysteines at these positions are important sites in cofilin for oxidation-mediated regulation of mitochondrial translocation [49]. Under these circumstances and concomitant with dephosphorylation of Serine 3 (S3), cofilin is prone to form cofilin-actin rods. ATP depletion is a major trigger for cofilin-actin rod formation at a stoichiometric ratio of 1:1 [88].

Cofilin-actin rods have also been suggested to possess protective properties under stress conditions [89]. They may protect against loss of the mitochondrial membrane potential and decline of cellular ATP level. Through rod formation, actin dynamics are alleviated, and energy can be used for other processes, enhancing the cellular resilience during stress exposure [90]. At later stages of the stress response, however, disrupted actin dynamics may counteract this positive energy-saving effect.

Mitochondrial translocation of cofilin was also observed in paradigms of apoptosis, as cofilin colocalization with mitochondria and subsequent release of cytochrome c is an early step in the cell death cascade [72,74]. It is the oxidized cofilin that is recruited to mitochondria in this case. The mechanism of cell death is described as oxidative cell death, more precisely, oxytosis and ferroptosis, at least in neurons [3]. ROS overproduction is induced by the formation of amyloid β (Aβ) plaques, which is promoted by the scaffolding protein RanBP9. This protein also delays clearance of cytosolic Ca^2+^ in a process involving the translocation of cofilin into mitochondria and oxidative mechanisms. This leads to neurodegenerative changes reminiscent of those seen in AD. RanBP9, cofilin, and Aβ mimic and potentiate each other in AD pathology [5].

The molecular mechanisms of cofilin-mediated distortion of mitochondrial function are beginning to unravel further. In neurons, cofilin depletion interferes with Drp1 accumulation at mitochondria [77] and cofilin-Drp1 interaction at the mitochondrial membrane and mitochondrial division [78]. At the same time, it was shown in mouse embryonic fibroblasts that maturation and activation of Drp1 oligomers at the mitochondrial surface, induced by cofilin depletion, increased mitochondrial fragmentation without impairing mitochondrial function in mouse embryonic fibroblasts [91]. To explain these discrepancies, the authors hypothesized that cofilin may control Drp1 accumulation in mitochondria and mitochondrial fission in an alternative manner during cell death and disease and during development. Dephosphorylation at S3 led to mitochondrial transactivation of cofilin and an interplay with Drp1 to enhance fragmentation of the organelle [77]. In yeast, it was demonstrated that cofilin mutants, deficient in actin binding, can enhance mitochondrial respiration, indicating that cofilin may also exert actin-independent effects on mitochondrial function [81].

## 5. Cofilin Mediated Mitochondrial Dysfunction During Tumorigenesis

ADF/cofilin family members are expressed at elevated levels in most tumor tissues and are thus regarded as oncogenes [22,92,93,94], as comprehensively reviewed recently [22]. Elevated levels of dephosphorylated cofilin were detected in different cancers.

The best studied mechanisms of cofilin involvement in tumorigenic processes are the cofilin-controlled turnover of cell surface receptors leading to increased oncogenic signaling; for epidermal growth factor receptor (EGFR), the cofilin-controlled actin turnover leading to increased migration of tumor cells has been best studied. In cofilin-knockout cells, it was demonstrated that the cell cycle was arrested in the G1 phase of the cell cycle, lamellipodia formation was impaired, and invasion and metastasis were reduced [95]. Another study found that the serum levels of cofilin immune complexes were significantly higher in pancreatic ductal adenocarcinoma patients than in healthy controls [96].

Cofilin localization at the mitochondria deserves more detailed investigation, especially its involvement in interaction with lipid droplets. C39, C80, C139, and C147 are the four important sites of cofilin for oxidation-mediated regulation of mitochondrial translocation [49,72] as well as its participation in the regulation of mitochondria-mediated apoptosis [97].

Mitochondrial homeostasis, controlled by mitochondrial membrane potential, mitochondrial fission, and mitochondrial autophagy processes, is one of the areas of focus in the development of cofilin-targeted drugs to target cancer cells and induce tumor cell apoptosis [98].

The factors controlling cofilin and Drp1 activities, being the main mediators of these processes, are the focus for the development of anti-cancer drugs. As PTEN/PI3K [99] and Src/Akt/mTOR [36] signaling pathways control the PP1/PP2A phosphatases that act upon cofilin [45], inhibitors of PI3K and Akt activities are used to inactivate cofilin. As a result, dephosphorylated cofilin translocates to the outer membrane of the mitochondria where it binds directly to F-actin and depolymerizes it, producing G-actin. The G-actin bound to cofilin enters the mitochondria and causes cytochrome c leakage into the cytoplasm [77]. This leads to activation of apoptosis-inducing proteases, starting with caspase 9 [99].

The interaction of Drp1 and cofilin is a target for drug development as it mediates mitochondrial fission. Drugs have been developed to target the PINK1/PARK2 pathway, which regulates the Drp1 phosphorylation and the GDP/GTP status of this GTPase [100,101]. The PINK1/PARK2 pathway is the key pathway that regulates mitochondrial autophagy [80]. As cofilin is involved in mitochondrial autophagy induction [79], drugs affecting the PINK1/PARK2 pathway are investigated with the prospect to suppress mitophagy in tumor cells.

## 6. Cofilin and Lipid Metabolism

**Figure 4:**
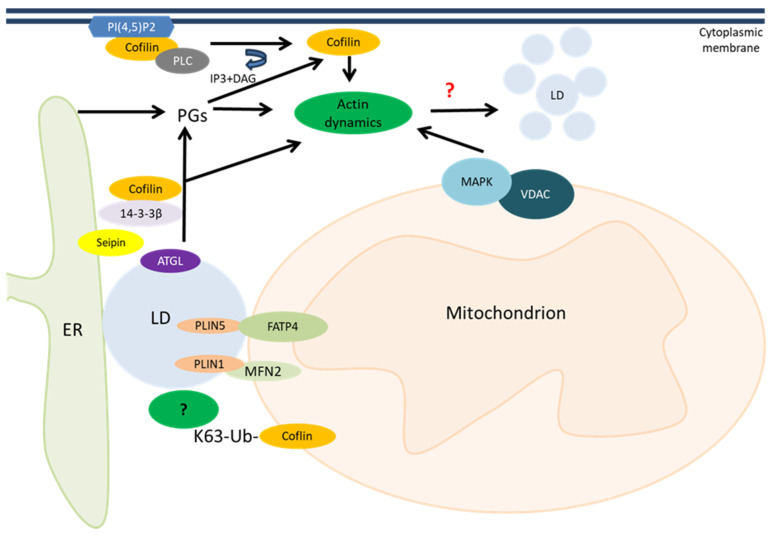
Cofilin is involved in lipid metabolism via prostaglandin (PG)-dependent and independent pathways, mainly affecting actin dynamics. Cofilin may be involved in the direct interaction between lipid droplets (LDs) and mitochondria. Cofilin activates the mitogen-activated protein kinase (MAPK) with a voltage-dependent anion channel (VDAC) located in the mitochondrial outer membrane [103]. Adipose triglyceride lipase (ATGL) releases arachidonic acid from triacylglycerols stored in lipid droplets, the substrate for PG synthesis [106]. Scaffolding protein 14-3-3β serves as a link between seipin (ER protein) and cofilin-mediated cytoskeleton reorganization [108]. Membrane-bound dephosphorylated cofilin can be activated through the cleavage of phosphatidylinositol 4,5-bisphosphate (PI(4,5)P_2_) by phospholipase C (PLC) [39].

Cofilin, in addition to affecting the morphology and movement of mitochondria, can affect their interorganelle interactions. One of these is the interaction of mitochondria with lipid droplets (LDs), which has recently gained attention [102]; this is also suggested by our preliminary data. In general, evidence is accumulating about the involvement of cofilin in lipid metabolism. As has been shown in yeast, cofilin-regulated actin dynamics lead to disruption of lipid homeostasis, accumulation of lipid droplets, and development of necrosis along with disruption of cell wall integrity and vacuole fragmentation. Briefly, cofilin activates the mitogen-activated protein kinase Slt complex with a voltage-dependent anion channel (VDAC) located in the mitochondrial outer membrane, which is called Porin 1 in yeast. This also provides evidence for a link between actin regulation and mitochondrial signaling [103] (Figure 4).

The members of the perilipin (PLIN) family PLIN1 and PLIN5 were shown to be involved in mitochondria-LD interactions. MFN2 specifically interacts with PLIN1 to form a protein complex tethering mitochondria to LDs. The acyl-CoA synthetase FATP4 (ACSVL4) was identified as a novel mitochondrial interactor of PLIN5 for channeling fatty acids from LDs to mitochondria and subsequent oxidation [104,105]. Adipose triglyceride lipase (ATGL) was shown to liberate arachidonic acid from triacylglycerols (TAGs) stored in LDs, providing the substrate for prostaglandin (PG) production by the endoplasmic reticulum (ER)-localized protein Pxt. Besides, arachidonic acid may be incorporated into phospholipids on LDs, ER, or other membranes [106]. Prostaglandin E2 (PGE2) was identified as an inhibitor of actin polymerization by activation of cofilin-1. This process is mediated by the protein phosphatase activity of PTEN [107].

Cofilin is involved in lipid storage signaling during adipogenesis by binding the cytoskeletal 14-3-3β protein in a spatiotemporal manner and mediating its interaction with the ER-resident protein seipin [108].

In addition, membrane-bound dephosphorylated cofilin can be activated through the cleavage of phosphatidylinositol 4,5-bisphosphate (PI(4,5)P_2_) by phospholipase C (PLC). As a result, active cofilin alters actin remodeling in the cytoplasm [39]. There is an intriguing possibility that ubiquitinated cofilin K63 may also affect lipid droplet levels by mediating their contacts with mitochondria through an as yet unknown cellular factor that ensures fatty acid entry into mitochondria [109]. In general, cofilin-1 depletion was reported to disrupt adipogenesis and lipid storage by inhibiting actin dynamics [110].

The involvement of lipid metabolism in tumor progression has been noted before and has been recently reviewed [111,112]. Moreover, the view that altered metabolism may be a driving force behind tumor initiation has now gained acceptance [112]. The tumor promoting role of altered lipid metabolism in cancers is now widely accepted as a favored means by which cancer cells may obtain energy, components for membranes, and a means of hijacking signaling molecules needed for proliferation, survival, invasion and metastasis, all of which may determine the response to cancer therapy.

Disturbed lipid metabolism in tumor cells is often characterized by the accumulation of lipid droplets. Lipid droplets are now recognized as cellular organelles and are the subject of current studies [113]. However, the functions of lipid droplets in cellular homeostasis and inflammatory signaling are far from being clear and require further research. This field of research is rapidly expanding and was already summarized in reviews over recent years [114,115,116].

The presence and function of LDs in the central nervous system has recently gained attention, especially in the context of neurodegeneration [117]. LDs are promising targets for novel investigations of neurological disease diagnosis and therapeutics. Further study on LDs and lipid metabolism will be essential in advancing the knowledge of cerebral metabolism, as well as the multifaceted etiologies of neurological disease. Therapeutic treatments could be targeted at restoring lipid balance, decreasing droplet levels, or improving other aspects of lipid metabolic pathways [118,119].

The dynamic contacts that tether lipid droplets to mitochondria are mediated through protein complexes, the identification of which is in urgent demand [102]. LDs not only bind organelles in a dynamic mode but also actively move [110] along actin networks and microtubules, apparently requiring the cofilin activity. This movement also requires tight regulation to be functional. The loss of proper contacts between mitochondria and lipid droplets is directly involved in tumorigenesis [113].

How contacts between lipid droplet and mitochondria affect the inflammatory response is an open question. Data obtained so far suggest that lipid droplets, as cell organelles and sources of lipid derivatives, can play opposite roles in inflammation, either promoting it or protecting against it, depending on cell type, cellular context, etc. [114,115]. Thus, it is a promising direction to study the involvement of cofilin in lipid metabolism and inflammatory processes.

The ubiquitous expression of cofilin and its involvement in many signaling pathways are reasons that viruses have highjacked cofilin signaling [38,120,121].

Deregulated expression and functions of cofilins are currently best studied in neurodegenerative pathologies and during tumorigenesis. However, a role of cofilin in metabolic disorders is now beginning to emerge, which is not surprising considering the control exercised by cofilin over mitochondrial traffic, mitochondrial division (fission), and mitochondrial membrane permeabilization.

Despite a great deal of knowledge about the functions of cofilin, some of its mechanisms of action remain a mystery and require further study. In this regard, it is relevant to elucidate the underlying mechanism of cofilin binding to mitochondria, and how cofilin may control contacts between mitochondria and other organelles.

## 7. Conclusions

Cofilin has emerged as a biomarker which is often targeted in different pathological conditions (Table 1). Specifically, its functional involvement in mitochondrial fission makes it interesting to investigate in connection with wound healing processes as it requires mitochondrial activity that is initiated by the fission processes.

All this motivates researchers to focus on the involvement of cofilin in metabolic processes occurring in different pathologies.

To elucidate the mechanics of altered metabolism in various pathologies, the proteome of the lipid droplet-mitochondria complex and, particularly, cofilin modified with K63 ubiquitin elongated chains deserves careful investigation in light of the proposed molecular mechanism linking lipid droplets and mitochondria [122]. An even more important task would be to reveal the dynamics of cofilin-mediated changes in mitochondrial metabolism.

The elucidation of inducible protein complexes on the mitochondrial membrane, at the interface between mitochondria and lipid droplets, and other preconditions for the formation of such complexes, can help create new drugs against malignant tumors.

**Table 1:** Functions of cofilin in the nerve and cancer cells.

Function	Nerve Cells	Cancer Cells	References
Actin dynamics and cytoskeleton remodeling	Regulates dendritic spine morphology via actin filament reorganization, synaptic plasticity, and cognitive functions	Drives cell migration and invasion via formation of lamellipodia/invadopodia	[22,123,124,125,126]
	Controls axon guidance and long-term potentiation (LTP)	Promotes metastasis by regulating cytoskeleton remodeling and EMT	
Mitochondrial dynamics	Oxidized cofilin translocates to mitochondria, initiating the release of cytochrome c, caspase activation, and apoptosis	Regulates cancer cell apoptosis depending on its activation state (phosphorylated/dephosphorylated)	[22,49,72,91]
	Mitochondrial fission mediated by both cofilin and Drp1	Drp1-dependent mitochondrial fission is a target for drug development	
Redox regulation	Serves as a cellular redox sensor. Oxidized cofilin forms rods with actin during ATP depletion (ischemia, Alzheimer’s disease)	Cancer cells modulate ROS to maintain cofilin in active/inactive states for invasion	[11,29]
	Cofilin-actin rods disrupt axonal transport and mitochondrial functions, impair synaptic function	Redox homeostasis affects cofilin-driven migration and survival	
Lipid signaling	PIP2 binding at the plasma membrane	Membrane PIP2 hydrolysis releases and activates cofilin	[8,127]
	PIP2 hydrolysis by PLC regulates cofilin activity and indirectly modulates synaptic vesicle trafficking	Supports proliferation and survival	
Lipid droplet dynamics	Potentially mediates LD-ER/mitochondria tethering via actin cytoskeleton	Potentially modulates actin-LD interaction	[106,112,118,128]
	LDs contribute to the pathogenesis of neurodegeneration	LDs promote cancer cell adaptation to oxidative stress and starvation	
Regulatory pathways	Phosphorylation/dephosphorylation (LIMK1/SSH etc.)	Phosphorylation/dephosphorylation (PTEN/PI3K and Src/Akt/mTOR signaling etc.)	[8,36,38,56]
	Ubiquitination	Ubiquitination	
Pathology	Neurodegeneration	Cancer	[22,42,44,76]

The involvement of cofilin in mitochondrial homeostasis, lipid metabolism, and the interaction between actin and microtubules of cytoskeleton, suggests that cofilin may play a role in the interactions of mitochondria with other organelles, such as lipid droplets. It would be interesting to investigate whether K63 ubiquitination of cofilin plays a functional role in this [11].

The functional effects of inducible cofilin translocations from the cytoplasm are gaining attention. It is now known that translocation of cofilin into mitochondria leads to a decrease in membrane potential [49]; if it translocates into the nucleus, it may affect DNA repair [129], and if it translocates to the cell surface, it may function as an autoantigen [130]. Thus, the role of cofilin in mediating mitochondrial contacts with lipid droplets deserves further investigation.

Deciphering how cofilin may control mitochondrial functions may reveal mechanisms that will help protect cells from unwanted signal rearrangement and metabolic changes and substantiate metabolically induced restoration of mitochondrial functions, i.e., through nutritional manipulation, used as an anti-cancer treatment.

The numerous controversies surrounding the involvement of cofilin in pathological processes have yet to be resolved. For example, cofilin is overexpressed in malignancies, but induction of cofilin activities and/or its increased expression is still suggested as a treatment of cancers or a means to inhibit migration of tumor cells [40]. However, cofilin expression in malignant tissues has also been reported as decreased [42]. Cells are arrested in G1 phase both when cofilin is knocked out and when its levels are elevated in confluent cells. These discrepancies may arise due to yet unknown mechanisms of cofilin expression regulation and/or use of different cell lines in the studies.

Recent advances in experimental techniques (mass spectrometry, microscopy, bioinformatics analysis) will certainly help to delineate the pleiotropic actions of cofilin, aid in identifying new post-translational modifications of cofilin, and further elucidate the role of known ones in the dynamic regulation of cellular homeostasis under stress. It will help to discover other proteins mediating mitochondria-lipid droplet contacts in addition to the very few that are known currently.

Drugs, peptides, or other substances targeting the critical amino acid residues of cofilin that control the interactions between mitochondria and lipid droplets might offer new potential therapeutic strategies for neurodegenerative disorders and tumors.

## Data Availability

Not applicable.

## Abbreviations

The following abbreviations are used in this manuscript:

AD	Alzheimer’s disease
ADF	Actin depolymerizing factor
AMPK	AMP-activated protein kinase
ATGL	Adipose triglyceride lipase
CIN	Chronophin
C	Cysteine
DIF-1	Differentiation-inducing factor 1
Drp1	Dynamin-related protein 1
EGFR	Epidermal growth factor receptor
EMT	Epithelial-mesenchymal transition
ER	Endoplasmic reticulum
F-actin	Filamentous actin
G-actin	Globular actin
K	Lysine
LD	Lipid droplet
LIMK	LIM kinase
LTP	Long-term potentiation
MFN2	Mitofusin 2
PD	Parkinson’s disease
PG	Prostaglandin
PI(4,5)P_2_	Phosphatidylinositol 4,5-bisphosphate
PLC	Phospholipase C
PLIN	Perilipin
PP1	Serine/threonine phosphatase type 1
PP2A	Serine/threonine phosphatase type 2A
PTEN	Phosphatase and tensin homolog
PTM	Post-translational modification
ROS	Reactive oxygen species
S	Serine
SSH	Slingshot phosphatase
T	Threonine
TESK	TES kinase
VDAC	Voltage-dependent anion channel
Y	Tyrosine
